# Mason: a JavaScript web site widget for visualizing and comparing annotated features in nucleotide or protein sequences

**DOI:** 10.1186/s13104-015-1009-z

**Published:** 2015-03-07

**Authors:** Daniel Jaschob, Trisha N Davis, Michael Riffle

**Affiliations:** Department of Biochemistry, University of Washington, UW Box 357350, 1705 NE Pacific St, Seattle, WA 98195-7350 USA; Department of Genome Sciences, University of Washington, UW Box 357350, 1705 NE Pacific St, Seattle, WA 98195-7350 USA

**Keywords:** Sequence annotation, Data visualization, Bioinformatics, Sequence feature annotation, Feature annotation, JavaScript, SVG

## Abstract

**Background:**

Sequence feature annotations (e.g., protein domain boundaries, binding sites, and secondary structure predictions) are an essential part of biological research. Annotations are widely used by scientists during research and experimental design, and are frequently the result of biological studies. A generalized and simple means of disseminating and visualizing these data via the web would be of value to the research community.

**Findings:**

Mason is a web site widget designed to visualize and compare annotated features of one or more nucleotide or protein sequence. Annotated features may be of virtually any type, ranging from annotating transcription binding sites or exons and introns in DNA to secondary structure or domain boundaries in proteins. Mason is simple to use and easy to integrate into web sites. Mason has a highly dynamic and configurable interface supporting multiple sets of annotations per sequence, overlapping regions, customization of interface and user-driven events (e.g., clicks and text to appear for tooltips). It is written purely in JavaScript and SVG, requiring no 3^rd^ party plugins or browser customization.

**Conclusions:**

Mason is a solution for dissemination of sequence annotation data on the web. It is highly flexible, customizable, simple to use, and is designed to be easily integrated into web sites. Mason is open source and freely available at https://github.com/yeastrc/mason.

## Introduction

Annotating regions or features within nucleotide and protein sequences (such as locations of binding sites, conserved residues, transmembrane regions, protein domain boundaries, or protein secondary structure) is a ubiquitous part of biological research. Previous annotations are an essential component of experimental design and interpretation, and new sequence annotations are often the goal of new studies—themselves becoming part of subsequent experimental design and interpretation in future studies. Given the growth of sequence annotation data and the importance of these data in research, it is becoming increasingly important to effectively disseminate and visualize these data. Of particular importance is the ability to merge separate sequence annotations into a single view that allows for the interpretation of new data in the context of known annotations.

Aligning and displaying multiple sequence annotations is already a core feature of genome browsers—software designed for navigating whole genomes and capable of visualizing a very wide array of annotations for genetic loci. Prominent examples of genome browsers include the UCSC genome browser [1], GBrowse [2], the Ensembl genome browser [3], and JBrowse [4]. While these tools are well-designed, mature, and feature rich; these tools are not designed to disseminate feature annotations for individual sequences outside the context of a broader genome. Other websites have developed web pages for displaying aligned feature annotations of individual protein sequences, including the UCSC Proteome Browser [5], the Protein Data Bank (PDB) [6], InterPro [7], WormBase [8], and the Saccharomyces Genome Database (SGD) [9]. While well-designed and informative, these views are optimized for the particular features they are displaying. Additionally, they are only available as parts of their respective web sites and not as a generalized distributable tool that may be integrated into other websites.

Recently, tools have started to emerge that are designed to visualize protein sequence feature annotations from any source on any web site. FeatureViewer [10], a component of BioJS [11], is a JavaScript library that uses SVG to render feature annotations. FeatureViewer is very customizable, but consequently complicated to set up. To simplify the setup, two extensions are provided: DasFeatureViewer and SimpleFeatureViewer. However, DasFeatureViewer requires the availability of a server-side Distributed Annotation System (DAS) resource and SimpleFeatureViewer has no support for overlapping feature annotations. pViz.js [12] is a JavaScript library that uses SVG and CSS to provide a dynamic interface for visualizing feature annotations in protein sequences. pViz is simpler to set up and requires no server-side component. However, pViz has only very basic support for overlapping annotations (annotations appearing on separate tracks). Additionally, pViz utilizes pre-defined CSS classes to assign different colors to different features, which limits pViz’s ability to achieve true-data driven coloring or shading schemes (such as shading based on confidences scores of the annotations), as all possible cases must be defined in advance. A comparison of the features offered by FeatureViewer, pViz and the work described here is presented in Table 1. These differences and their significance are further explored in the context of actual applications in “Findings”, under “Current Implementations.”

**Table 1:** **Comparison of main features between Mason and pViz.js**

**Feature**	**Mason**	**pViz.js**	**FeatureViewer**
Simple, standalone component	✓	✓	‡
Dynamic interface	✓	✓	✓
Click and mouseover events	✓	✓	✓
No browser plugins required	✓	✓	✓
Data-driven coloring and shading	✓	†	✓
Optimized for many overlapping annotations	✓		
Row-level summary bars	✓		
Zooming		✓	✓
Customizable annotation shapes		✓	✓
Export Image			✓

†pViz.js, by default, supports color customization via CSS, which requires pre-defined color definitions for classes of annotations in advance and is not amenable to true data-driven coloring schemes where color may indicate any possible value. ‡DasFeatureViewer extension of FeatureViewer requires a server-side DAS data source (SimpleFeatureViewer does not).

Here we present Mason, a generalized web site module designed to display sequence feature annotations on any web site. Mason aligns and displays many sequence feature annotations in a single, dynamic view and is particularly well-suited for many overlapping annotations. Mason is independent of any specific source or type of annotation and is highly customizable, supporting true data-driven tooltips, click events, and coloring. It is written purely in JavaScript and SVG, requiring no 3^rd^ party plugins. Mason is designed to be simple to use, easy to set up, and requires no server-side component. Mason is open-source and freely available at https://github.com/yeastrc/mason.

## Findings

### Web component

Mason is implemented using only standard World Wide Web technologies: JavaScript, HTML, and Scalable Vector Graphics (SVG). Consequently, no 3^rd^ party plugins are required for modern web browsers. Mason is cross-platform and has been tested with current versions of Chrome, Firefox, Safari, and Internet Explorer running on Windows, Linux, MacOS, and iOS. Mason has no server-side component other than the availability of the data to be displayed. Mason makes use of the jQuery (http://jquery.com/), svg.js (http://www.svgjs.com/), wz_tooltip.js (http://www.walterzorn.com/), and Modernizr (http://modernizr.com/) JavaScript libraries—all of which are available at the mason GitHub site at https://github.com/yeastrc/mason.

### Software architecture

Mason is designed to be flexible and customizable with regard to type and source of sequence annotations. All of the code that is independent of a specific type of data (such as building the viewer itself or detecting user events) is contained in the Mason core. All of the code that is specific to a particular type of data is passed into the core when the viewer is instantiated as a set of JavaScript callback functions that adhere to a specific interface. This set of callback functions, which may be collectively considered a module, is then used by the core to provide custom behavior for a specific instance of the Mason viewer.

The Mason core expects the input data to be provided at the time of instantiation and for that data to adhere to a specific Javascript object structure. This provides Mason with independence from any particular source of data and allows the code for processing the data to be a part of the Mason core, but requires that the source data be converted to this structure before being passed to Mason. Further customization is achieved by providing simple customization parameters to the Mason core at the time of instantiation. These parameters include items such as row heights, border colors, or font sizes.

Full implementation details, including examples and documentation of the interfaces for callback functions, input data format, and the customization options are provided at the Mason GitHub site at https://github.com/yeastrc/mason. Additionally, this site includes several pre-built modules for common sources of sequence annotations. These are discussed in more detail in the Results section.

### Installation

The simplest method for installing Mason is by using one of the pre-built modules that supports the output of a specific sequence annotation program (described below) or by using the more-flexible generic JavaScript Object Notation (JSON) module that may be used for data from any source. Along with the pre-built modules, the generic JSON module requires no knowledge of JavaScript to implement and requires no server-side component. It only requires that the data be formatted as JSON text using a relatively simple pre-defined schema (available at our web site). The generic JSON module supports tooltips, linking annotations to external URLs, expanding overlapping annotations, and row-level coloring. To install the generic JSON module, first include the necessary JavaScript files on the page using standard HTML. Then, create a DIV on the page with the pre-defined class (“generic-json-mason-viewer”) that references the location of the data, such as:



The data will be read in from the indicated file location and a Mason viewer will be automatically created at the location of the DIV. (Note: because of web browser security models, the JSON file must be accessed via a web server and that must be the same web server address as the HTML file referencing it.) Alternatively, the text in  above may be present within the page, itself, by leaving out the  attribute and assigning the “masonData” variable equal to the text contents of the file inside of a < script > element. For full documentation, including the syntax of the JSON, examples, and download files for the generic JSON viewer, visit the Mason demo page at http://www.yeastrc.org/mason/.

To apply Mason to sequence annotation data that is beyond the scope of the pre-built modules, it is necessary to write code to convert the annotations to the expected input format and to write a series of callback functions to customize the look and behavior of Mason (see “Software Architecture, above). Note: that a working proficiency with JavaScript is necessary for this step. Once the data is formatted and the callback functions are written, Mason may be instantiated on the page using the JavaScript function call:



Where  is the location on the page to build the viewer (jQuery variable),  includes the data to be displayed,  includes configuration parameters, and  is an object containing the customized callback functions that constitute a module for a given type of sequence annotation. Note that multiple Mason viewers may be added to the same page by making multiple calls to .

Detailed documentation for installation, the input data format, configuration parameters, and the callback functions are available at the Mason GitHub site at https://github.com/yeastrc/mason.

### Graphical user interface

#### Basic functionality

**Figure 1:**
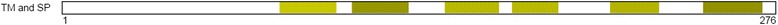
**An example of a single mason viewer with a single row.** This is a depiction of predicated transmembrane regions in a protein. The protein’s sequence is graphically represented as a row with the N-terminus on the left and the C-terminus on the right. The predicted transmembrane regions, shaded yellow, are mapped onto this representation based on the positions of their start and end residues.

**Figure 2:**
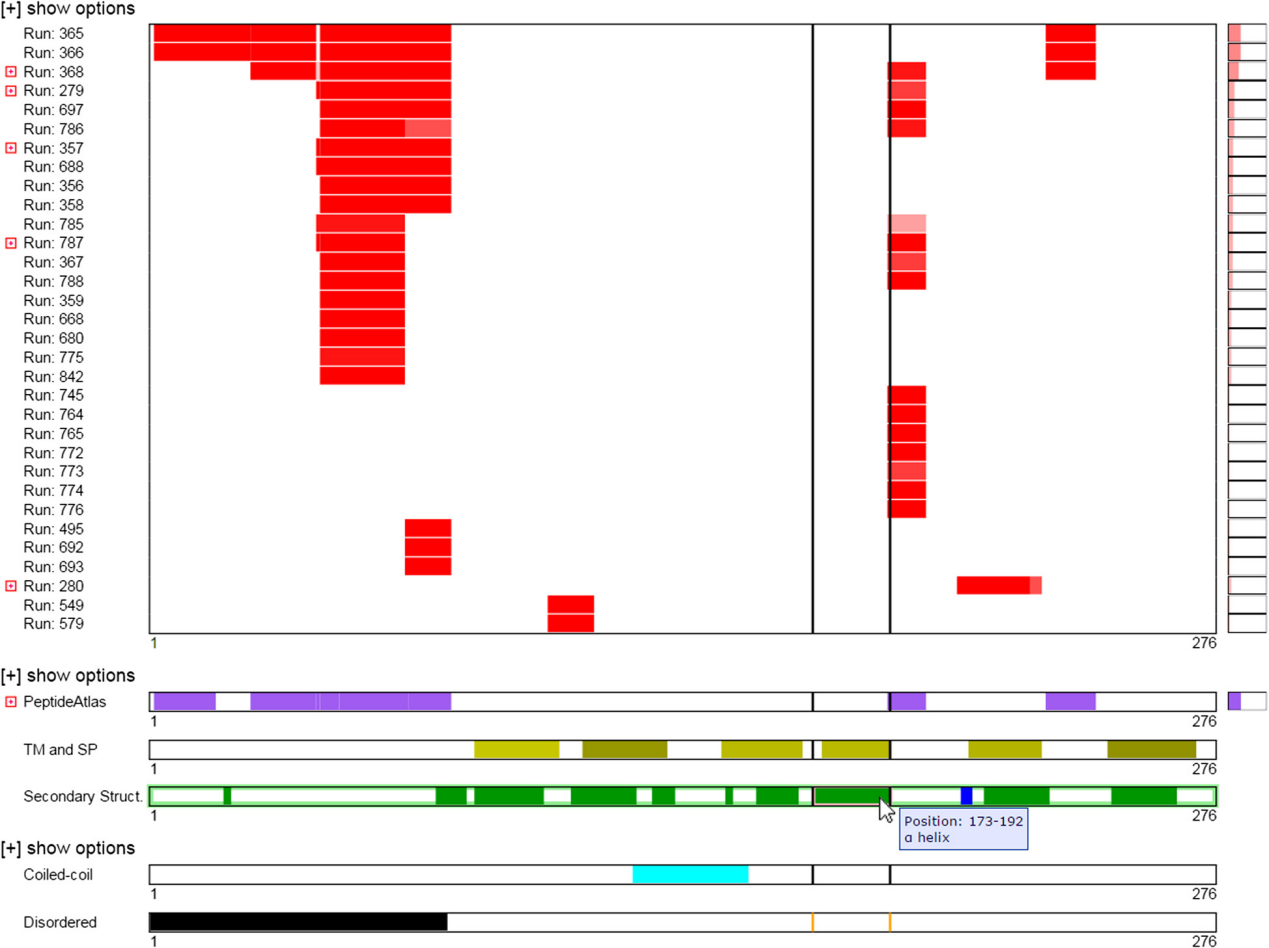
**An illustration of the more advanced features of the Mason viewer.** In this example, five mason viewers are loaded on a single web page, each depicting a different type of sequence annotation data. The top-most viewer, with red blocks, illustrates a single mason viewer loaded with multiple rows. Each row depicts the peptide coverage for this protein from multiple mass spectrometry proteomics experiments. The summary bar on the right-hand side of the rows indicates the total protein sequence coverage in each experiment. The viewer with the purple bars indicates the peptide coverage for the same protein from the PeptideAtlas [13] resource of proteomics experiments. The viewer with yellow bars indicates predicted transmembrane regions. The viewer with green and blue bars depicts the predicted secondary structure for that protein. The viewer with cyan bars indicates predicted coiled-coils in that sequence. And, the viewer with black bars indicates the predicted disordered regions in that protein. The vertical lines present in the same position in all viewers represent the boundaries of the feature annotation the mouse pointer is currently on. And the tooltip present next to the mouse pointer is presenting information about that annotated feature.

The Mason viewer graphically represents a sequence horizontally, with position 1 on the left and the final position on the right. Each set of feature annotations is represented as a separate row, where each annotation includes a starting and ending position in the sequence. These annotations are represented as blocks in that row that start and end at the specified positions (Figure 1). Mason is capable of displaying multiple rows of annotations per viewer, which is meant to display multiple sets of annotations of the same type from separate sources (e.g., sets of secondary structure predictions from different programs or protein coverage from multiple proteomics experiments) (Figure 2). Because sequence positions are consistent between multiple rows in the Mason viewer, the positions of the annotations may be directly compared between the different rows. Additionally, multiple Mason viewers containing data of different types may be available on the same page (e.g., one viewer for secondary structure predictions and one viewer for disordered regions) (Figure 2). The positions in the sequences between different viewers also line up and may also be directly compared. Furthermore, Mason is aware of multiple instances of the Mason viewer on the same page, and provides a visual indication of how annotations in distinct viewers line up when the user moves their mouse arrow over an annotation of interest (or tap on mobile devices) (Figure 2).

### Overlapping feature annotations

**Figure 3:**
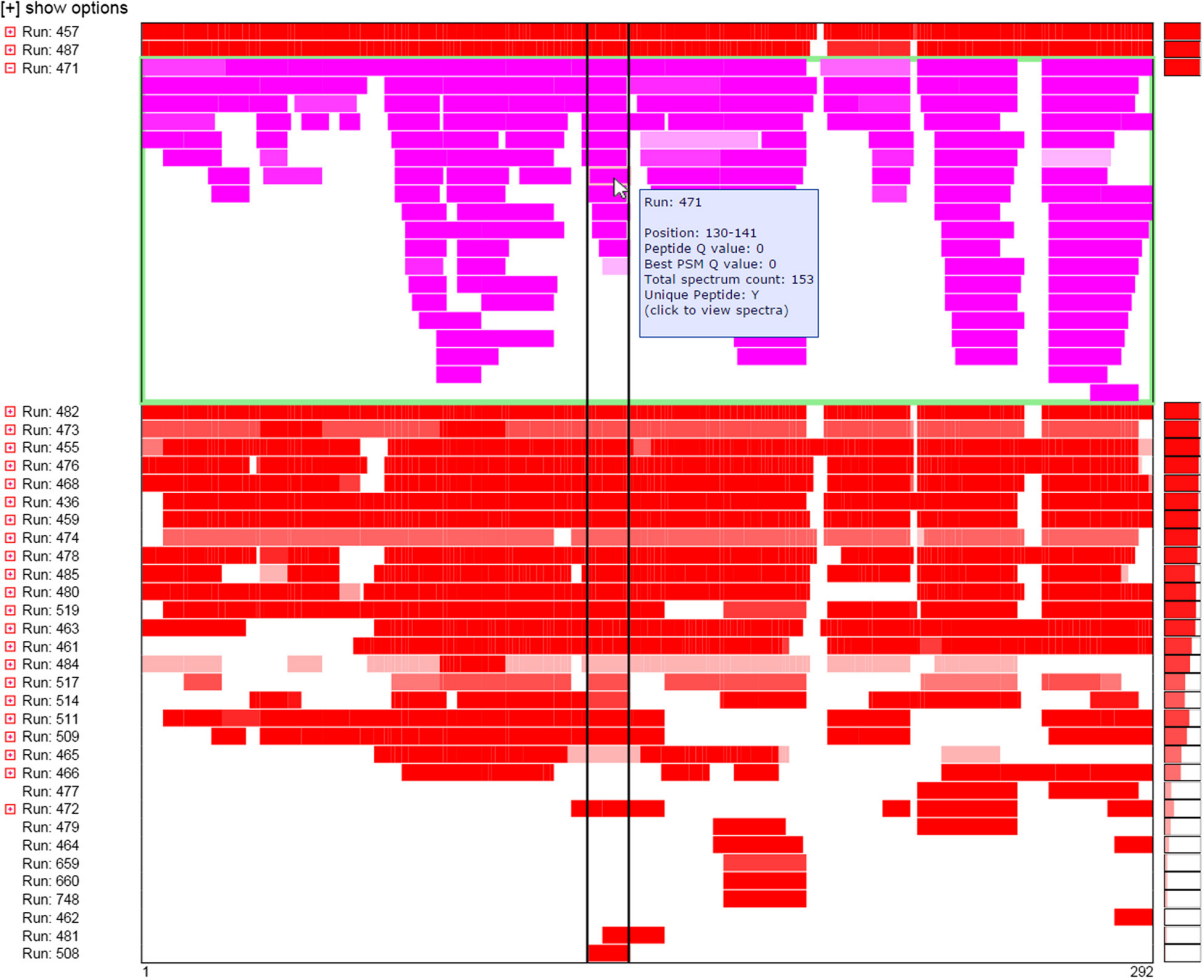
**An illustration of how Mason handles overlapping feature annotations.** The small box with the plus sign to the left of the row label indicates that overlapping feature annotations are present in that row. Clicking that box will expand the row such that all distinct feature annotations are displayed. In this case, a single mason viewer with multiple rows is shown. The user has clicked the box next to “Run: 471” and the row expanded to show all distinct annotations for that row in shades of magenta. The mouse pointer has been placed over a distinct annotation, resulting in the display of vertical lines showing the boundaries of that annotation across all rows and a tooltip describing that annotation.

Feature annotations may sometimes overlap in the sequence. For example, annotation A may describe positions 2–10 and annotation B may describe positions 8-19—creating overlapping annotations for positions 8–10. Visually, this will appear as a single block from positions 2–19; however, a clickable icon will appear to the left of the row label that indicates overlapping annotations are present. When click, that row will expand such that overlapping features are displayed in multiple rows, ensuring all distinct annotated features may be displayed (Figure 3).

### Tooltips and click events

Text to appear in a tooltip when the user mouses-over (or taps) on any annotated feature may be defined in a callback function passed into the Mason viewer creator (see Implementation). Examples include displaying the starting and ending positions and the confidence scores associated with the annotation. Likewise, the result of clicking (or double tapping) on any of the annotated features may be similarly defined via another callback function. This may be useful as a means for users to click through to another web page with more information about the specific annotation.

### Colors and shading

The color of the blocks in the Mason viewer may be customized via a callback function that has access to the data associated with the annotations. This enables a very broad range of capabilities regarding data visualization. Coloring schemes may range from simple (all blocks are the same color) to more sophisticated schemes that use shading to indicate annotation confidence scores or separate colors to indicate annotation properties (such as different colors for an alpha-helix or beta-sheets in secondary structure predictions).

### Lines noting positions of interest

**Figure 4:**
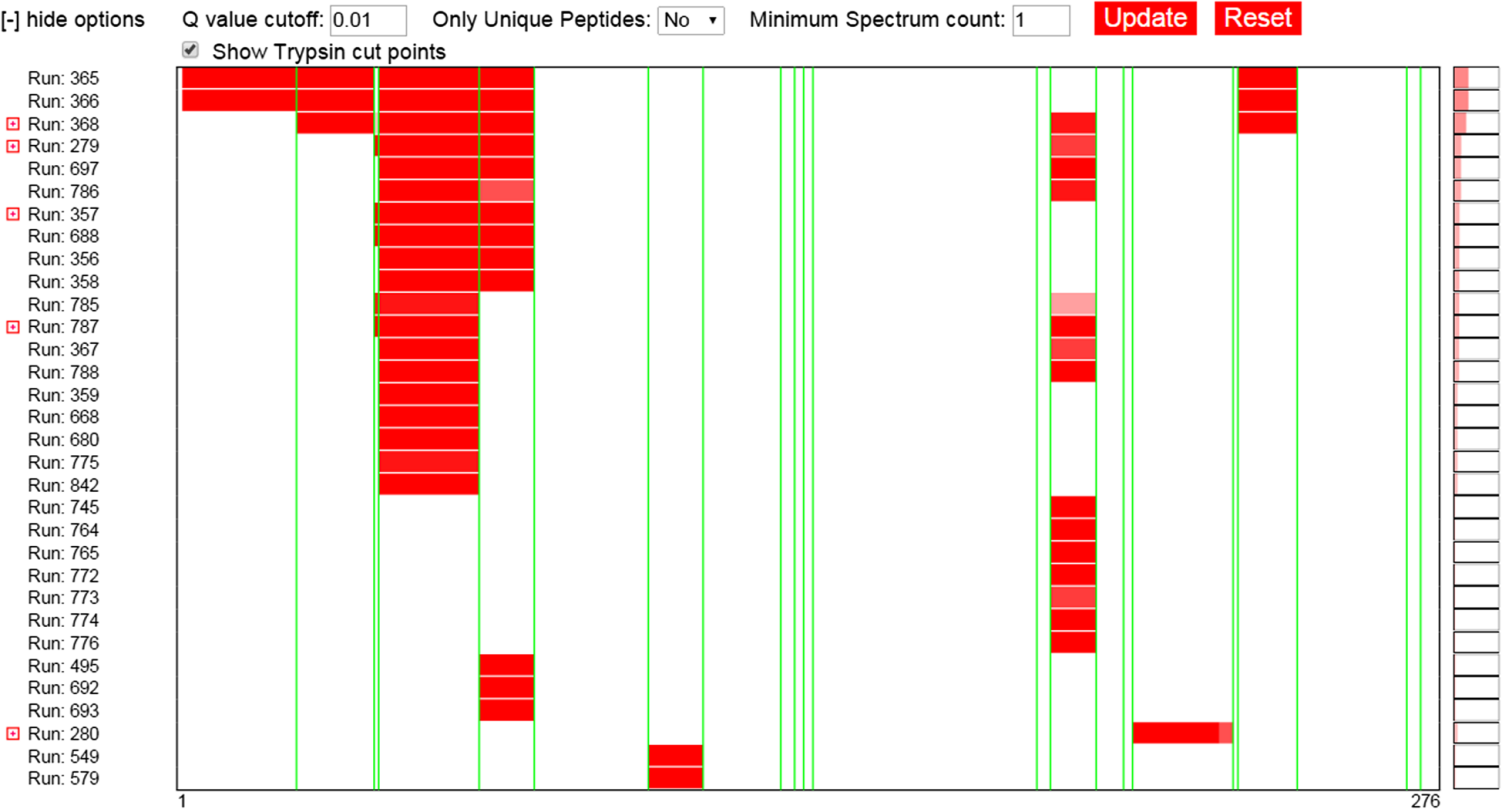
**An illustration of lines noting positions of interest and how an options menu can be used with the Mason viewer.** In this example, a single Mason viewer depicting the peptide coverage for a single protein from multiple mass spectrometry proteomics experiments is shown. In the experiments, the proteins have been digested with trypsin and the green vertical lines represent positions in the protein’s sequence that contain the trypsin cut motif. The expectation is that all peptides should be terminated on both ends by a trypsin cut site. The presence of the green lines is controlled via an options menu, which is not itself a part of Mason, but can interact with the Mason viewer via Javascript function calls. In this case, checking the “Show Trypsin cut points” checkbox toggles the green vertical lines on and off by calling functions in the Mason core.

Mason may also display vertical lines at specific positions in the rows to note positions of interest that aid in interpretation of the data. Examples would include noting cleavage sites in DNA sequences or trypsin cut sites in protein sequence (Figure 4). The positions to draw lines is passed into the Mason creator, the color of the lines are defined via callback functions, and the visibility of the lines may be toggled via a simple function call to the Mason viewer.

### Summary bars

Mason may optionally show a summary bar on the right-hand side of the rows to visually indicate some type of summary statistic associated with the entire row of sequence annotations. Examples including showing protein quantitation data or protein sequence coverage for a given mass spectrometry run. Multiple rows containing summary bars effectively provide a horizontal bar graph for comparing summary statistics between rows. Custom colors, shading, tooltips, and click handlers may be defined for the summary bars using callback functions.

### Current implementations

**Figure 5:**
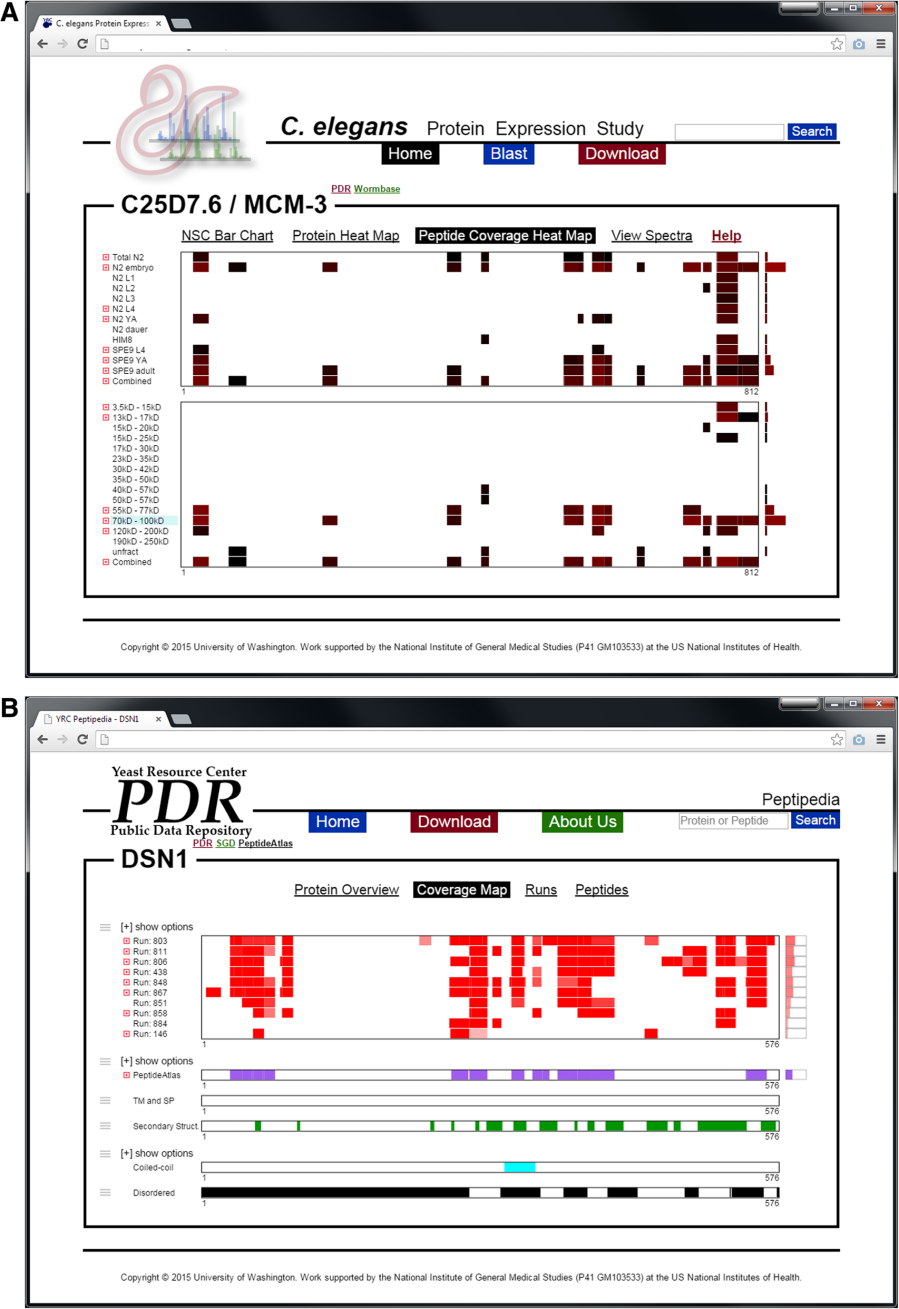
**Screenshots from two implementations of the Mason viewer on data-driven web applications. (A)** Mason is used to show protein sequence coverage, relative protein abundance, and relative peptide abundance across many conditions. The top viewer compares the data across multiple developmental stages of the model organism *C. elegans*, and the bottom viewer compares the data across multiple mass fractions. The summary bar to the right of the viewer indicates overall relative protein abundance (as compared between conditions in the respective viewers). The protein and peptide abundance is shown using shades of red, where black represents the least abundance and bright red represents the most. The rows with red boxes to the left of the labels may be expanded to disambiguate the observed peptides. Each disambiguated peptide may be clicked on to view the underlying mass spectrometry data. **(B)** Mason is used to show protein sequence coverage (viewer with red bars) among many mass spectrometry runs. The bars to the right represent total sequence coverage for the protein in the respective run. Shades of red in the rows indicate the quality of scores the peptide identification received, and the shade of red in the row level summary bar serves as a secondary indication of protein sequence coverage. Each row with the red box to the left of the label may be expanded to disambiguate overlapping peptides, and each peptide may be moused-over to view summary information and clicked on to view underlying mass spectrometry data. The other viewers (purple, green, cyan, and black) show annotations for this protein from other sources.

The Mason viewer has been integrated into two upcoming (not yet published) large-scale proteomics data resources (Figure 5). In the first case (Figure 5A), Mason is used to visualize the relative abundance of a protein and the relative abundance of the individual peptides used to identify that protein across many different conditions. This implementation of Mason makes use of the summary bar feature (to the right of the rows) to show overall relative protein abundance, makes use of data-driven coloring and shading to provide an indicator for relative abundances of the peptides, and makes use of Mason’s ability to disambiguate overlapping annotations to show relative abundances of distinct peptides that were identified and to provide links for viewing the underlying mass spectrometry data collected for each peptide. FeatureViewer and pViz.js would not be suitable solutions for this visualization, as the row level summaries and dynamic disambiguation of overlapping annotations are essential aspects of this view of the data. Additionally, coloring and shading that describe underlying values in the data (such as quality or quantity of identifications) would be difficult to accomplish by pre-defining classes of colors using CSS, which is the default coloring model used by pViz.js.

In the second case (Figure 5B), Mason is again used to visualize the coverage of a protein in many different conditions—but in this case, those conditions are separate proteomics experiments where the protein was identified. The top viewer in the figure visualizes the coverage of this protein in many different runs, where the shading of the red blocks indicates the strength of the identification, and the row-level summary bars to the right indicate the overall protein coverage in that run. In this type of data, identifying overlapping peptides for a protein in an experiment is very common, so the ability to handle many overlapping annotations for the protein is essential to effectively disseminating the data. Attempting to show all disambiguated peptides from all runs at once in multiple tracks would result in a much more cluttered and non-informative view. To provide context, the remaining viewers on the page display annotations for this protein from other sources, and makes use of Mason’s ability to communicate between instances of the viewer to show precisely how annotations in one viewer map to the others.

### Pre-built examples

Several pre-built code examples are available for displaying data from common sources of sequence annotations. Working demos and downloads are available at http://www.yeastrc.org/mason/.

### Generic JSON module

The Mason site includes code for reading and displaying data formatted as JSON adhering to a simplified schema (available on the web site). This module is suitable for providing a simple view of sequence annotation data from nearly any source, especially data that has many overlapping annotations. This module supports overlapping features, tooltips, links to external URLs, and row-level coloring.

### Transmembrane and signal peptides

The Mason site includes code for displaying transmembrane and signal peptide predictions from the Philius prediction server [14]. The code accepts a protein sequence directly, submits this to the Philius prediction server, and displays the results in the newly-built Mason viewer. Only the protein sequence is required, and there is no need to install or run Philius on the part of the web site operator.

### Secondary structure

The Mason site includes code for displaying predicted protein secondary structure as generated by the psipred program [15]. This is accomplished by pointing the code to the URL for a .ss2 file (PSIPRED VFORMAT) that is generated by the psipred program—the code for accessing the data and converting it to JSON is provided. Consequently, psipred must be run in advance and the resulting file made available on a web server.

### Coiled-coil regions

The Mason site includes code for displaying predicted coiled-coil regions generated by the Paircoil2 program [16]. This is accomplished by pointing the code to the URL for a .pc2 file that is generated by the Paircoil2 program—the code for accessing the data and converting it to JSON is provided. Consequently, Paircoil2 must be run in advance and the resulting file made available on a web server. This module also includes a custom options menu that allows the user to filter the data based on the P-score generated by Paircoil2.

### Disordered regions

The Mason site includes code for displaying predicted disordered regions generated by the DISOPRED program [17]. This is accomplished by pointing the code to the URL for a .diso file that is generated by the DISOPRED program—the code for accessing the data and converting it to JSON is provided. Consequently, DISOPRED must be run in advance and the resulting file made available on a web server.

## Conclusions

The Mason viewer is a generalized, flexible, and portable web site module capable of displaying DNA or protein sequence annotations for single sequences. Mason is designed to be integrated with existing 3^rd^ party web applications, though some familiarity with JavaScript is required. Although Mason has a highly dynamic interface, it uses only standard web technologies, requires no 3^rd^ party web browser plugins, and is designed to be simple-to-use and intuitive for end users. Mason is open-source and is freely available at the Mason GitHub site at https://github.com/yeastrc/mason. The site includes extensive documentation and examples, including pre-built code for displaying sequence annotations from several existing sources.

### Availability and requirements

- Project name: Mason
- Project home page: https://github.com/yeastrc/mason
- Operating system(s): Platform independent
- Programming language: JavaScript, HTML, SVG
- Other requirements: None
- License: Apache 2.0
- Any restrictions to use by non-academics: None

## Abbreviations

CSS: Cascading style sheets
DAS: Distributed annotation system
GUI: Graphical user interface
HTML: Hypertext markup language
JSON: JavaScript object notation
SVG: Scalable Vector Graphics
